# Contact lens physical properties and lipid deposition in a novel characterized artificial tear solution

**Published:** 2011-12-24

**Authors:** Holly Lorentz, Miriam Heynen, Lise M.M. Kay, Claudia Yvette Dominici, Warda Khan, Wendy W.S. Ng, Lyndon Jones

**Affiliations:** Centre for Contact Lens Research, School of Optometry, University of Waterloo, Waterloo, Ontario, Canada

## Abstract

**Purpose:**

To characterize various properties of a physiologically-relevant artificial tear solution (ATS) containing a range of tear film components within a complex salt solution, and to measure contact lens parameters and lipid deposition of a variety of contact lens materials after incubation in this ATS.

**Methods:**

A complex ATS was developed that contains a range of salts, proteins, lipids, mucin, and other tear film constituents in tear-film relevant concentrations. This ATS was tested to confirm that its pH, osmolality, surface tension, and homogeneity are similar to human tears and remain so throughout the material incubation process, for up to 4 weeks. To confirm that silicone hydrogel and conventional hydrogel contact lens materials do not alter in physical characteristics beyond what is allowed by the International Organization for Standardization (ISO) 18369–2. The diameter, center thickness, and calculated base curve were measured for five different lens materials directly out of the blister pack, after a rinse in saline and then following a two week incubation in the modified ATS. To test the ATS and the effect of its composition on lipid deposition, two lens materials were incubated in the ATS and a modified version for several time points. Both ATS solutions contained trace amounts of carbon-14 cholesterol and phosphatidylcholine, such that deposition of these specific lipids could be quantified using standard methods.

**Results:**

This ATS is a complex mixture that remains stable at physiologically relevant pH (7.3–7.6), osmolality (304–306 mmol/kg), surface tension (40–46 dynes/cm) and homogeneity over an incubation period of three weeks or more. The physical parameters of the lenses tested showed no changes beyond that allowed by the ISO guidelines. Incubations with the ATS found that balafilcon A lenses deposit significantly more cholesterol and phosphatidylcholine than omafilcon A lenses (p<0.05) and that removing lactoferrin and immunoglobulin G from the ATS can significantly decrease the mass of lipid deposited.

**Conclusions:**

This paper describes a novel complex artificial tear solution specially designed for in-vial incubation of contact lens materials. This solution was stable and did not adversely affect the physical parameters of the soft contact lenses incubated within it and showed that lipid deposition was responsive to changes in ATS composition.

## Introduction

In vitro biomaterial models have been used extensively to analyze surface interactions that occur with an implanted medical device and their surroundings [1-5]. Contact lenses are similar to an implant in that they are a biomaterial that is exposed to a very complex biologic environment, in some cases more complex than permanently implanted biomaterials, such as a hip or knee replacement. Unlike these biomaterials, contact lenses are exposed to a continuously changing tear film composition and structure, induced by continuous blinking and drying of the lens surface, changes in environmental surroundings, systemic diseases, medications, alcohol consumption and diet [6-9].

The composition of the human tear film is complex and contains several layers, including a glycocalyx-like mucin layer covering the corneal epithelium, an aqueous layer rich in proteins, salts and electrolytes, and a lipid layer divided into both a polar and non-polar lipid component [10-12]. Although this layered tear film model is still favored, it is now believed that this structure is not as compartmentalized as previously thought and that the components from each layer can be found throughout the entire tear film [13-17]. Soft contact lens materials, once inserted into the eye, lie in the middle of this tear film structure and are known to readily adsorb many different tear film components, including lipids, proteins, and mucins [18-27].

Building an in vitro model to examine deposition of tear film components onto contact lens materials would allow for systematic and structured analysis of tear film interactions. These models could then be used to analyze various lens materials and their affinity for different tear film components, the conformation of proteins on contact lens materials, the exploration of tear film component interactions and competition, and the effectiveness of contact lens cleaning solutions to remove such deposits. These types of experiments would be difficult, if not impossible, to conduct in a controlled manner using in vivo or ex vivo studies. Therefore, in vitro models examining these interactions and processes can provide pertinent information to further our understanding of the ever growing field of contact lens material science.

In vitro models have many benefits over in-eye clinical studies. They allow for analysis of specific variables without the use of human or animal testing, the variables are easily and tightly controlled in laboratory settings, many different analysis techniques can be used that otherwise would not be available using in vivo or ex vivo based studies, allow for the examination of both simple and complex models, and lastly in vitro studies tend to require less financial support and time to conduct, since participant remuneration and ethics approval are not required.

Although in vitro models can never fully mimic the complex nature of human contact lens wear, they can be designed to be physiologically relevant and help understand the basic tear film interactions that occur. Many early in vitro contact lens deposition models involved incubating contact lens materials in a simple saline solution with one tear film component, such as a single protein or lipid [18,24,28-31]. This model is very simplistic and is not indicative of what is found in the human tear film. It is clear that there is a relative dearth of information on contact lens in vitro models, especially for lipid deposition.

More recently, researchers have started to increase the complexity of the artificial tear solutions used to mimic the tear film. Mirejovsky et al. [32] was the first to report on the use of a complex artificial tear film that contained a range of salts, proteins, and lipids. Artificial tear solutions used in in vitro studies must contain physiologically relevant components, maintain physiologically relevant solution properties and must not change the contact lens parameters during incubation, as alterations in these parameters can cause changes in the contact lens dimensions themselves. The contact lenses may swell/shrink, thicken/thin, or experience a change in their base curve if an inappropriate solution is used. These lens parameter changes could alter the deposition pattern and lens interactions with tear film components. If in vitro contact lens deposition models are to mimic human contact lens wear, then the artificial tear solutions used must be more complex than a single component system. Recent work from our laboratory has shown that an in vitro incubation solution consisting of a mixture of lipids, proteins, mucins and buffers is significantly different to that obtained in an in vitro model which uses single lipids alone [33]. In this study, we wanted to explore how sensitive the lipid deposition was to smaller changes in solution, such as adding or removing individual components.

Our laboratory has characterized a complex physiologically relevant artificial tear solution (ATS) designed for in vitro vial-enclosed incubation experiments. This solution has been tested to determine if the solution and contact lens parameters remain stable throughout contact lens incubation. Although this solution does not contain all of the individual human tear film components, it does contain a broad representation of the most abundant lipids, proteins, mucin, salts, and inorganics that are present.

## Methods

### The ATS composition

ATS preparation required four main steps. These included preparation of the complex salt solution, lipid stock solution, adding lipids to the salt solution, and addition of the proteins and mucin to complete the solution.

#### The complex salt solution

The first step in making an ATS was the preparation of a complex salt solution (CSS). The composition of the CSS, which is used as the base of the ATS, is shown in Table 1. These specific salts and their relative concentrations are based on literature values [32,34-36]. All CSS components were ACS grade and purchased from Sigma (Oakville, ON). The individual components were measured on an analytical balance and sequentially added to the desired volume of MilliQ water in the order that they are listed in table. Once all of the components had been added, ProClin 300 (Sigma, Oakville, ON), a preservative and antimicrobial agent, was added to the system. The use of ProClin 300 allows for incubation at 37 °C for prolonged periods of time with no fear of microbial contamination. After all the ingredients were added, the pH was approximately 7.15 and the osmolality was 305 mmol/kg. When the CSS was left at room temperature for three or more days it equilibrated naturally to the desired pH of 7.4, which is the typical pH of the human tear film [37]. However, if the solution was to be used immediately then purging with nitrogen gas equilibrated the solution to the desired pH much faster.

**Table 1:** Artificial tear solution complex salt solution components [35-38].

**Salt component**	**Molecular formula**	**mM**
Sodium chloride	NaCl	90.0
Potassium chloride	KCl	16.0
Sodium citrate	Na_3_C_6_H_5_O_7_	1.5
Glucose	C_6_H_12_O_6_	0.2
Urea	(NH_2_)_2_CO	1.2
Calcium chloride	CaCl_2_	0.5
Sodium carbonate	Na_2_CO_3_	12.0
Potassium hydrogen carbonate	KHCO_3_	3.0
Sodium phosphate dibasic	Na_2_HPO_4_	24.0
Hydrochloric acid (10 molar)	HCl	26.0
ProClin 300 (Supelco 48912-U)		0.2 µl/ 1l
MilliQ Water		

#### Concentrated lipid stock solution

The next step in the ATS preparation was to make a concentrated lipid stock. Here, a 2,000× concentrated lipid stock solution (LSS) was made to help facilitate dissolving the pure lipids into the CSS. Lipids, especially non-polar lipids, do not naturally dissolve into aqueous solutions, so dissolving them first into a solution of 1 hexane: 1 ether and then adding an aliquot of the hexane/ether LSS to the CSS helps facilitate the incorporation of lipids. To make a LSS, pure lipids were warmed up to room temperature and weighed out using an analytical balance (solid lipids) or pipetted using a positive displacement pipette (liquid lipids). The concentrated LSS was placed in an amber vial, sealed with Parafilm^®^ (VWR, Mississauga, ON), wrapped in foil and stored at −20 °C until required. Table 2 shows the lipids used in the ATS, their characteristics, the lipid stock concentration and final ATS concentration used for each lipid. All pure lipids were purchased through Sigma (Oakville, ON). The lipids used in this ATS were chosen specifically so that a broad range of human tear film lipids were represented and their concentrations were chosen based on human tear film concentrations, artificial tear solution literature values, and lipid solubility in aqueous solutions [28,32,38-40].

**Table 2:** Molecular and experimental details of the specific lipids used for all lipid incubation solutions [28,35,40-42].

**Pqarameters**	**Triolein**	**Cholesterol**	**Oleic acid**	**Oleic acid methyl ester**	**Cholesteryl oleate**	**Phosphatidyl choline**
Lipid type	Triglyceride	Sterol	Fatty acid	Fatty ester	Cholesteryl ester	Phospholipid
Formula	C_57_H_104_O_6_	C_27_H_46_O	C_18_H_34_O_2_	C_19_H_36_O_2_	C_45_H_78_O_2_	C_42_H_82_NO_8_P
Molecular Weight (g/mol)	885.5	386.7	282.5	296.5	651	760.1
Lipid Stock Concentration (mg/ml)	32.0	3.6	3.6	24.0	48.0	1.0
Final ATS Concentration (mg/ml)	0.016	0.0018	0.0018	0.012	0.024	0.0005

#### Lipid artificial tear solution

The next step in making an ATS was to make the lipid artificial tear solution (LTS). This was accomplished by removing the LSS from the freezer and allowing it to warm up to room temperature in a dry dark place. The desired volume of room temperature CSS was placed into a glass septum jar and the required volume of LSS was added to the CSS. The cap was screwed onto the septum jar and the whole jar was placed into an ultra-sonic bath that was warmed to 37 °C. Two syringes were pierced through the septum, one large blunt syringe was placed into the solution and one smaller syringe was left sitting in the air space of the septum jar. The large syringe was connected to a nitrogen tank and the small syringe remained open to air to act as a vent. The LTS was sonicated at 90 W and purged with nitrogen gas at a pressure of 3 psi until the LSS was fully incorporated into the CSS and the odour of hexane:ether had dissipated. The LTS was now complete.

#### Incorporation of proteins and mucin to complete preparation of the ATS

The last step in preparing the ATS was the addition of proteins and mucin. The specific proteins and mucin used and their concentrations in the final ATS are outlined in Table 3 and are based on literature values of the human tear film, literature ATS concentrations, and based on the cost of the component, as in the case of lactoferrin and IgG [32,41-48]. All proteins and mucin were purchased from Sigma. Bovine and hen-egg proteins were chosen for use in this ATS due to their cost and their similarities to human proteins in molecular weight, pI, amino acid chain length, and number of charged residues. The proteins and mucin were weighed out on an analytical balance and added to the LTS while stirring. When all components were incorporated fully, the complete ATS was sonicated at 37 °C for a maximum of 5 min, to prevent destruction of the proteins [49].

**Table 3:** Protein and mucin concentrations and details in ATS [35,43-50].

**Proteins**	**Molecular weight (kDa)**	**Concentration (mg/ml)**	**Sigma product number**
Bovine albumin	66.4	0.20	A7888
Hen egg lysozyme	14.3	1.90	L6876
Bovine submaxillary mucin	3×10^5^ to 4×10^7^	0.15	M3895
Bovine colostrum lactoferrin	83.1	1.80	L4765
Bovine immunoglobulin G	161	0.02	I5506

### Solution properties

#### pH and osmolality

To test the consistency of the ATS’s pH and osmolality during in vitro incubations, a 28 day study was performed. Clear borosilicate glass 6 mL vials were half filled with freshly made ATS with a starting pH of 7.35 and an osmolality of 305 mmol/kg. Vials were closed with PTFE-sealed screw caps, further sealed with Parafilm^®^ and incubated at 37 °C for six different time points including: 1, 3, 7, 14, 21, and 28 days in triplicate. On the specific days, the vials were opened and the pH was measured using the SympHony SB20 pH meter (VWR, Mississauga, ON) and the osmolality was measured using the Wescor “Vapro” Vapor Pressure Osmometer 5520 (Discovery Diagnostics, Claremont, ON).

#### Surface tension and homogeneity of ATS

To test the surface tension and liposome homogeneity of the solution a 3.5 week study was conducted. Fresh ATS was made and tested for its surface tension and homogeneity and then the ATS was incubated for 3.5 weeks at 37 °C and tested again for the two parameters. Surface tension was measured using the Wilhelmy balance (CAHN Instruments, Madison, WI) using a platinum ring and the homogeneity of the solution was tested by staining liposomes in the ATS with Nile Red. To stain with Nile Red, the Nile Red was dissolved in acetone at 1 mg/ml, then 1 µl of the Nile Red solution was added to 100 µl of the test solution in a micro-centrifuge tube and shaken so the two components were well mixed [32]. Then 20 µl of the Nile Red test solution was then pipetted onto a slide (prewashed with methanol), and a coverslip was placed on top. The sample was then examined and photographed on the microscope at 10× and 40× magnifications using a green light filter. Samples of the complex salt solution and artificial tear solution were analyzed at several points in the preparation process and compared with the solution after 3.5 weeks of incubation. The distribution and diameter of the liposomes was analyzed for each sample.

### Lens parameters

Five contact lens materials were tested in triplicate: Acuvue^®^ 2 (etafilcon A; Vistakon, Jacksonville, FL), Proclear^®^ (omafilcon A; CooperVision, Pleasanton, CA), Acuvue^®^ OASYS™ (senofilcon A; Vistakon), Biofinity^®^ (comfilcon A; CooperVision), PureVision™ (balafilcon A; Bausch & Lomb, Rochester, NY). The material characteristics of all contact lens materials can be found in Table 4 and Table 5. All lens materials tested had a spherical power of −3.00 diopters (D) and had an approximate base curve of 8.6±0.2 mm. The individual lenses were measured on three separate occasions: out of the blister pack, after 40 h of soaking in CSS, and after a 2 weeks incubation at 37 °C in the ATS previously described. The center thickness was measured using a Rehder Development Co. E.T.-1 (Castro Valley, CA) and the diameter and sagittal height (Sag) of each lens was measured using the Optimec Soft Contact Lens Analyzer (Malvern, UK). The base curve was then calculated from the diameter and sagittal height. The data were analyzed using Statistica 9 using paired *t*-tests. The contact lens parameter measurements were taken so that comparisons could be made between the three parameters tested and was not meant to assess the contact lens parameter variability from their specified package dimensions.

**Table 4:** Conventional hydrogel contact lens material characteristics.

**Material type**	**Conventional hydrogel**	
USAN	Etafilcon A	Omafilcon A
Proprietary name	Acuvue®2	Proclear®
Manufacturer	Johnson & Johnson	CooperVision
Power (D)	−3.00	−3.00
Base Curve (mm)	8.7	8.6
Diameter (mm)	14.0	14.2
Monomers	HEMA, MA	HEMA, PhC
Surface Modification	None	None
Oxygen Transmissibility (×10^−9^)	31.0	52.3
Water Content	58%	62%
FDA class	Group IV	Group II

USAN: United States adopted name; HEMA (poly-2-hydroxyethyl methacrylate); MA (methacrylic acid); PhC (phosphorylcholine).

**Table 5:** Silicone hydrogel contact lens material characteristics.

**Material Type**	**Silicone Hydrogel**		
USAN	Senofilcon A	Comfilcon A	Balafilcon A
Proprietary name	Acuvue® OASYS™	Biofinity®	PureVision™
Manufacturer	Johnson & Johnson	CooperVision	Bausch & Lomb
Power (D)	−3.00	−3.00	−3.00
Base curve (mm)	8.4	8.6	8.6
Diameter (mm)	14.0	14.0	14.0
Centre thickness (mm) −3.00D	0.07	0.08	0.09
Monomers	mPDMS, DMA, HEMA, siloxane macromer, EGDMA, PVP	M3U, FM0411M, HOB, IBM, NVP, TAIC, VMA	NVP, TPVC, NVA, PBVC
Surface modification	PVP as an internal wetting agent	None	Plasma oxidation
Oxygen transmissibility (×10^−9^)	147	160	110
Modulus (MPa)	0.7	0.75	1.1
Water content	38%	48%	36%
FDA class	Group I	Group I	Group III

USAN: United States adopted name; DMA (N,N-dimethylacrylamide); EGDMA (ethyleneglycol dimethacrylate); FM0411M (2-ethyl [2-[(2-methylprop-2-enoyl)oxy]ethyl]carbamate); HEMA (poly-2-hydroxyethyl methacrylate); HOB ((2RS)-2-hydroxybutyl 2-methylprop-2-enoate); IBM (Isobornyl methacrylate); M3U (α-[[3-(2-[[2-(methacryloyloxy)ethyl] carbamoyloxy]ethoxy)propyl]dimethylsilyl]-ω-[3-(2-[[2-(methacryloyloxy)ethyl] carbamoyloxy]ethoxy)propyl]poly([oxy[(methyl) [3-[ω-methylpoly(oxyethylene)oxy]propyl]silylene] /[oxy[(methyl)(3,3,3-trifluoropropyl)]silylene]/oxy (dimethylsilylene)])); mPDMS (monofunctional polydimethylsiloxane); NVA *N*-vinyl aminobutyric acid; NVP (N-vinyl pyrrolidone); PBVC (poly[dimethysiloxy] di [silylbutanol] bis[vinyl carbamate]); PVP (poly(vinylpyrrolidone)); TAIC (1,3,5-triprop-2-enyl-1,3,5-triazine-2,4,6(1H,3H,5H)-trione); TPVC (tris-(trimethylsiloxysilyl) propylvinyl carbamate); VMA (N-Vinyl-N-methylacetamide).

### Lipid deposition

**Figure 1:**
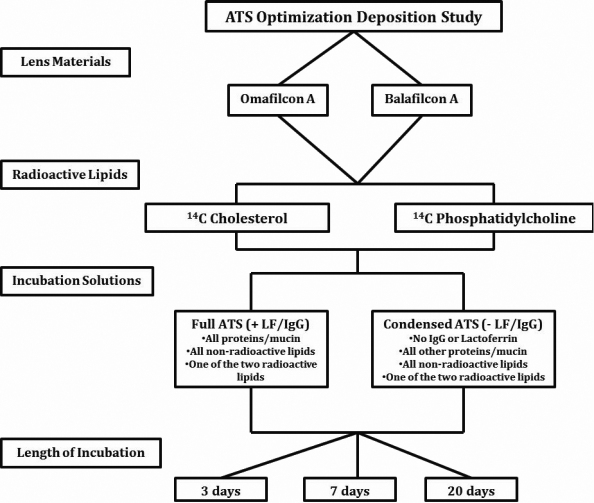
Lipid deposition study outline using the artificial tear solution (ATS) and two radiolabeled lipids.

As the last step of the ATS characterization process, the ATS was examined for its lipid deposition using a simple radioactive experiment previously developed by our laboratory. In this experiment, omafilcon A and balafilcon A lens materials were incubated in two different ATS solutions for three different time periods, as outlined in Figure 1. The first ATS solution composition was identical to the ATS described above (+LF/IgG) and the second ATS solution was a slightly simpler version with lactoferrin (LF) and immunoglobulin G (IgG) removed (- LF/IgG). To facilitate sensitive quantification of lipid deposition, both ATS solutions were prepared by adding a small aliquot of one of two radiolabelled lipids (Table 6); ^14^C-cholesterol or ^14^C-phosphatidylcholine. Lenses (n=3) were then incubated in each solution for 3, 7, and 20 days.

**Table 6:** Radioactive lipid characteristics.

**Lipid**	**Radiolabel**	**Molecular weight (g/mol)**	**Supplier**
Cholesterol [C]	4-^14^C	386.6	Perkin-Elmer
L-α-DiPalmitoyl-Phosphatidylcholine [PC]	DiPalmitoyl-1-^14^C	734.0	Perkin-Elmer

At the end of the incubation period, each lens was rinsed twice in saline and blotted on lens paper. The lenses were then placed in 20 ml glass scintillation vials with 2 ml of 2:1 chloroform:methanol extraction solution and were incubated for three hours each at 37 °C while shaking on an orbital shaker. Each lens was extracted in this way on two separate occasions and both extracts were pooled together in the same vial.

The extract vials were dried completely using nitrogen evaporation at 37 °C. All samples were re-suspended in 1 ml of chloroform, sonicated for one min, and 10 ml of Ultima Gold F scintillation cocktail (Perkin-Elmer, Woodbridge, ON) was added. The vials were submitted for liquid scintillation beta counting. Standard lipid samples were prepared and all data were analyzed and quantified using standard calibration curves.

## Results

### pH and osmolality

When examining the stability of pH and osmolality of the ATS it was found that pH ranged from 7.35 to 7.49 and osmolality ranged from 305.0 to 303.7 mmol/kg, over the 28 days of incubation.

### Surface tension and homogeneity of ATS

After the complex salt solution and ATS preparation was complete, several aliquots of each solution were stained with Nile Red examined microscopically at 200×-400× and photographed. Following a three week in-vial incubation, ATS aliquots were once again stained and photographed. Following staining with Nile Red, the CSS samples had no visible liposomes present in its solution, as expected. However, both ATS samples, freshly made and post incubation solutions, showed similar distribution and sizes of liposomes stained by the Nile Red. The liposomes present in both ATS solutions ranged in size from 6 to 20 µm, with average sizes around 12 µm. Therefore, no discernible differences were found in fresh versus incubated ATS solutions in terms of its homogeneity.

The surface tension of the freshly prepared ATS was 51.5±0.38 dynes/cm and following the 25 days of incubation the surface tension fell to 45.05±1.25 dynes/cm. This is an average change of −6.46±1.30 dynes/cm.

### Lens parameters

**Figure 2:**
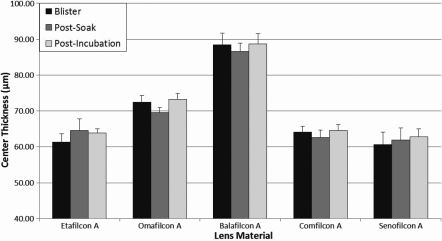
Average center thickness of all studied lens materials as measured directly from the blister pack, after a saline soak, and following 14 days incubation in ATS.

The center thickness of each lens material measured out of blister pack, following a saline soak, and after ATS incubation at 37 °C for two weeks can be graphically seen in Figure 2. One statistically significant difference was seen when analyzing the difference between the blister pack and post-incubation conditions. Omafilcon A lenses experienced a 1.0% average increase in center thickness following two week incubation in ATS. These changes in center thickness would not correlate to any significant clinically relevant changes in vivo.

**Figure 3:**
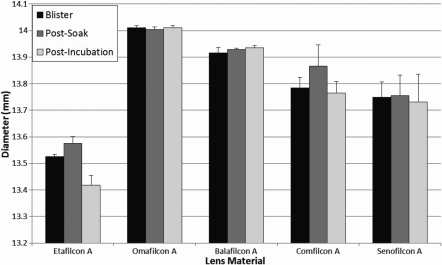
Average contact lens diameter of all studied materials as measured directly from the blister pack, after a saline soak, and following 14 days incubation in ATS.

The average contact lens diameter results measured out of blister pack, following a CSS soak, and following a two week incubation in ATS can been seen in Figure 3. Only etafilcon A had a statistically significant change in diameter following incubation in ATS, where the average diameter decreased by 0.81%. These changes in diameter are not considered to correlate to any clinically significant changes in vivo.

**Figure 4:**
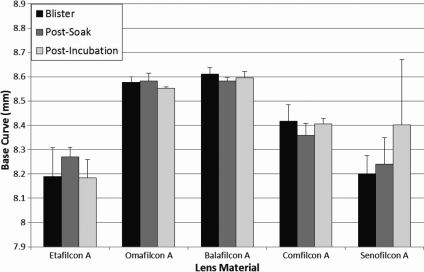
Average contact lens base curve of all studied materials as measured directly from the blister pack, after a saline soak, and following 14 days incubation in ATS.

Average base curve results for each contact lens material after each lens treatment are displayed in Figure 4. No statistically significant differences were seen when comparing the blister pack measurements to the post-incubation in ATS measurements for any lens material.

### Contact lens lipid deposition

**Figure 5:**
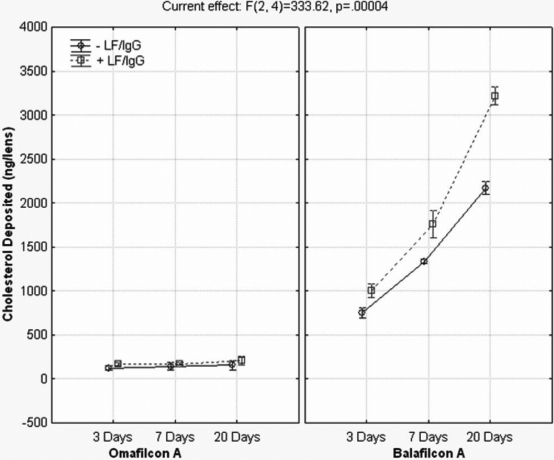
Cholesterol deposition with and without lactoferrin and immunoglobulin G for omafilcon A and balafilcon A. -LF/IgG=no lactoferrin and immunoglobulin G in the ATS. +LF/IgG=lactoferrin and immunoglobulin G were present in the ATS.

**Figure 6:**
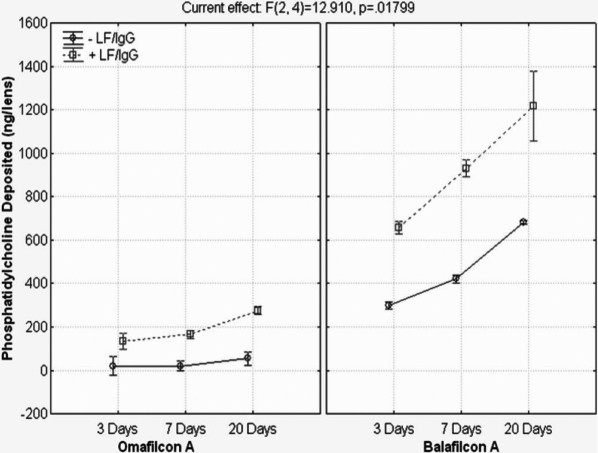
Phosphatidylcholine deposition with and without lactoferrin and immunoglobulin G for omafilcon A and balafilcon A. -LF/IgG=no lactoferrin and immunoglobulin G in the ATS. +LF/IgG=lactoferrin and immunoglobulin G were present in the ATS.

The results of the radioactive cholesterol (C) and phosphatidylcholine (PC) kinetic uptake with and without the presence of lactoferrin and IgG can be seen in Figure 5 and Figure 6. As seen in the figures below, the silicone hydrogel lens material deposited more than the conventional hydrogel lens and that more cholesterol was deposited than phosphatidylcholine. The lipid uptake for all lens materials, especially the silicone hydrogels, was continuous throughout the 20 day period, with no plateau. The presence of lactoferrin and IgG in the ATS correlated with a statistically significant increase in cholesterol and PC deposition for balafilcon A at every time point (p≤0.001). Cholesterol deposition on omafilcon A in the presence of LF/IgG was greater than without, however the trend was not statistically significant for any time point (p>0.05). However, PC deposition on omafilcon A did show statistically significant increases in the presence of LF/IgG for every time point (p≤0.008). Overall, there were statistically significant differences in the entire repeated measures ANOVA model, including all the variables and variable interactions for each lipid tested, as seen in Table 7 and Table 8.

**Table 7:** Cholesterol repeated measures ANOVA results.

**Variables**	**SS**	**DF**	**MSq**	**F**	**p**
Time	5255742	2	2627871	2774	<0.0001
±LF/IgG	851579	1	851579	1739	0.0006
Lens	21480765	1	21480765	24367	<0.0001
Time * ±LF/IgG	266177	2	133089	196	0.0001
Time * Lens	4865540	2	2432770	1506	<0.0001
±LF/IgG * Lens	634230	1	634230	794	0.0013
Time * ±LF/IgG * Lens	254090	2	127045	334	<0.0001
Error	1523	4	381		

SS=sum of squares, DF=degrees of freedom, MSq=mean square, F=F statistic, p=probability.

**Table 8:** Phosphatidylcholine repeated measures ANOVA results.

**Variables**	**SS**	**DF**	**MSq**	**F**	**p**
Time	476975	2	238488	737.92	<0.0001
±LF/IgG	889902	1	889902	1468.12	0.0007
Lens	3127668	1	3127668	2588.36	0.0004
Time * ±LF/IgG	30717	2	15358	21.80	0.0071
Time * Lens	221962	2	110981	424.64	<0.0001
±LF/IgG * Lens	212945	1	212945	440.59	0.0023
Time * ±LF/IgG * Lens	5516	2	2758	12.91	0.0180
Error	855	4	214		

SS=sum of squares, DF=degrees of freedom, MSq=mean square, F=F statistic, p=probability.

## Discussion

In the creation of an in vitro model designed to analyze the dynamics of tear film interactions on a contact lens surface, the development of an appropriate artificial tear solution that is both physiologically relevant and stable is imperative. A handful of papers has been published using in vitro experimental models to examine contact lenses, their deposition and their tear film interactions. Many of these papers have used very simple in vitro solution models with single components for investigation, such as a single lipid or protein. These individual component model systems have been regularly used for the past 25 years and are continually being used. In the mid-1980s, Castillo et al. [50] used lysozyme incubation solutions dissolved in a phosphate buffered saline (PBS) to examine conformational changes that occur on PHEMA materials fabricated using different methods via ATR-FTIR. Garrett et al. [24] and several studies from Jones et al. [18,29,30] used lysozyme or lactoferrin-only solutions in PBS for radiochemical studies examining lysozyme or lactoferrin adsorption and conformation onto various contact lens materials. Similar to proteins, there are several papers using single lipid in vitro systems, including Carney and colleagues work in 2008, where they examined kinetic uptake of lipid onto various contact lens materials using fluorescently labeled cholesterol and phosphatidylethanolamine solutions independently [28]. Most recently, Pucker et al. [31] published a similar paper examining the uptake of cholesterol oleate and phosphatidylcholine separately in an undisclosed buffer solution. In most of these publications, a PBS solution with a single lipid or protein is used; however in many of the papers there is no information about the specific composition or concentrations of the PBS itself. Since there is no standardized composition of PBS, many of these papers are lacking important information regarding the ATS used.

There are several experimental papers where moderately complex in vitro artificial tear solutions were used. These solutions are mixtures of proteins or lipids dissolved into a saline base. Castillo et al. [51] and Bohnert et al. [52] both used an ATS which contained a mixture of several proteins dissolved into a saline solution to examine protein adsorption and conformation onto contact lens materials. Ho and Hlady examined lipid deposition using a mixture of several lipids dissolved into a more complex mixture of salts [53]. In each of these three examples, lipids and protein components were not mixed together within the ATS and there was no incorporation of mucin.

Recent work from our laboratory [33] and past work from Bontempo and Rapp [23,54] have found a dramatic difference in the amount of lipids and proteins deposited onto conventional and silicone hydrogel contact lens materials from an ATS of different complexities. Single component systems, moderately complex systems (no mixing of lipids and proteins together) and complex multiple lipid and protein systems have different deposition behaviors. Although simpler systems can be useful for particular experimental models, they are unsuitable to mimic human contact lens wear deposition and tear film interactions, due to their lack of complexity.

Papers have been published introducing more complex in vitro artificial tear solutions. The first of these papers was by Mirejovsky et al. [32] in 1991, where lipids, proteins, mucin, and a variety of salts were all incorporated to form a complex tear solution. Mirejovsky’s ATS contains a range of different proteins, lipids from different classification groups, and a non-physiologic biochemical buffer. It was more complex than many of the past solutions and the first to more accurately mimic human tear fluid with individualized concentrations for each component. Since the introduction of Mirejovsky’s ATS, several other research groups have begun using a more complex ATS, including Prager and Quintana [25,44], Bontempo and Rapp [54,55], and Iwata et al. [56]. Prager and Quintana’s [25,44] solution had the same protein portion as the Mirejovsky ATS and the lipid portion was similar, but instead of using a specialized blend of salts, Prager and Quintana used a Hank’s Balanced Salt solution as their saline base. The Bontempo and Rapp [54,55] ATS incorporated five tear film lipids, all incorporated in the same concentration, three tear film proteins, all incorporated in the same concentration, and a 0.9% saline base. The most recent solution of note is the one used by Iwata et al. [56] This solution used a mixture of four lipids, three proteins and a simplistic saline base [56].

It is common in in vitro ATS deposition models that the ATS is a homogenous composition with the proteins, lipids, and mucin mixed together throughout the solution. In other words, the solution is not in the layered biophysical structure as it is in the natural tear film. This is for several reasons; first, in-vial static aqueous incubations are not conducive to a lamellar structure, as the contact lens would not be exposed to all of the tear film components as they are in human contact lens wear. The blinking action, tear film mixing, tear film thinning and the eventual tear film breaking that occurs in human contact lens wear exposes the lens to all layers and components of the tear film. The second reason for using a homogenous non-layered incubation solution is because this model is simpler to execute and has similar deposited masses of tear film components as ex vivo examined lenses [57,58]. Therefore, the biophysical arrangement of the ATS does not impact deposition to the same extent as the interactions that occur between the contact lens and tear film components. Therefore, even though the ATS structure is not necessarily identical to human tear film structure, it is still known to be a good model for deposition and tear film interaction research. Future models will incorporate a layered tear film analog and incorporate air exposure, mimicking the inter-blink period.

With the modified ATS solution introduced in this paper, we have tried to combine all of the necessary complexity by incorporating a variety of lipids, proteins, mucin, salts and also other prevalent tear film components such as physiologic buffers, glucose and urea, all within a stable system specially designed for in-vial incubations. All of these previously published variations on an ATS are indeed a great improvement over the more simplistic solutions based, primarily, on saline with a few added components. However, none of the papers described has shown the stability of the reported solutions, especially in terms of their pH and osmolality during the various contact lens incubations. Work in our laboratory during the development of this ATS clearly demonstrated the importance of reduced carbonates and increased phosphates in the complex saline solution, which was used as the base solution, to maintain pH and osmolality over time.

It is known that the pH and osmolality of a stable human tear film is 6.6–7.8 [37] and 305 mmol/kg [59], respectively, and that the surface tension of tears is 40–46 dynes/cm [60]. Therefore, we contend that the model ATS with the specific complex salt solution introduced in this paper is a suitable physical and chemical representation of the human tear film. The complex salt solution introduced in this paper was specially designed and extensively tested to confirm its stability. Many different combinations and concentrations of salts and physiologic buffers were tested, however many of the test solutions did not remain stable in pH or osmolality over time. This was especially true for solutions with higher concentrations of carbonates, as carbonates tend to react with carbon dioxide in the air and therefore easily lead to a change in pH, especially if vials are not tightly sealed. This process was exacerbated when the ATS was incubated in plastic vials, instead of glass. All plastic vials tested, including low-density polyethylene, high-density polyethylene, super polyethylene, and Teflon-coated plastic vials all have intrinsic gas permeability and therefore the pH and osmolality of the ATS was constantly changing. Therefore, the final stable physiologically relevant complex salt solution modified by our laboratory contained only biologic buffers and a slightly reduced concentration of carbonates. This solution was specifically designed for closed in-vial incubations within borosilicate glass vials with screw caps with PTFE liners that are sealed with Parafilm^®^, so that ATS pH and osmolality remained stable throughout the incubation periods.

In all of these papers on in vitro model systems, only one of them has mentioned the lens parameter changes that occur upon incubation. Pucker et al. [31] admit that due to the incorporation of chloroform in their incubation solution, the lens materials do indeed swell. Most of the other systems do not have this chloroform addition and the extra solvents such as hexane that may be present from the use of a lipid stock are evaporated before lens incubation. None of the other papers has reported measuring the diameter, center thickness or base curve before incubation and following incubation in their ATS to know if the composition of the ATS is causing lens parameter changes beyond that which is considered allowable by the FDA. Contact lenses and their cleaning solutions are tightly regulated so that contact lens parameter changes do not occur. According to the ISO tolerance guidelines [61], contact lens materials are only allowed to change by ±0.20 mm in diameter and base curve, and by approximately ±18 µm in center thickness, depending on the specific lens material, during cleaning or contact lens wear. Swelling, stretching, shrinking and curvature changes could all induce power changes, fitting changes, and comfort issues for the contact lens wearer. In an in vitro experiment, these changes can affect contact lens deposition and interactions with tear film components so that the contact lenses no longer react naturally to their surroundings.

In this experiment, the diameter, center thickness, and base curve of all contact lens materials were measured directly after removing them from the blister pack, following a soak in CSS, and after two weeks of incubation in the artificial tear solution described. The diameter, base curve and center thickness measurements all showed no clinically significant changes following incubation in the ATS and no parameter changes were found beyond what is allowed by 2006 ISO 18369–2 tolerance guidelines [61]. In a few instances, statistically significant changes in lens parameters were found between the blister pack measurements and following incubation in the ATS, however these changes were still well within ISO tolerances.

As the final step in the development of this ATS, the ATS was tested for its ability to deposit lipid onto both a conventional and silicone hydrogel contact lens material. Omafilcon A and balafilcon A lenses were chosen for the experiment, as previous research has shown that conventional hydrogels tend to deposit low amounts of lipid, whereas silicone hydrogel lenses, especially balafilcon A, are known to be more lipophilic and more likely to deposit lipid [28,56,62]. Cholesterol and phosphatidylcholine were chosen for examination using a radiochemical experiment. Radiochemical experiments have been widely used in biomaterials research [63-68] including contact lens research, especially protein deposition research [18,24,25,29,30,69]. It has been shown to be a very sensitive, repeatable and reliable method of analysis and thus was chosen for this experiment. Cholesterol was selected as a representative non-polar lipid as it has been widely cited to be one of the most prevalent deposited lipids [57,62,70-72] and phosphatidylcholine was chosen as a polar lipid species, due to its presence in the tear film [11,73,74].

The results of the deposition experiment clearly showed that lipid deposition, especially on balafilcon A lenses, tend to continually deposit without a plateau effect throughout the 20 day incubation period, that the specific composition of the ATS will have a large impact on the deposition pattern for lipids, and that cholesterol tends to deposit more than phosphatidylcholine. Bontempo and Rapp [54] previously examined the impact that ATS composition has on lipid and protein deposition for conventional hydrogel lenses, but to date nothing has been published on silicone hydrogel lens materials.

This research supports the notion that the specific composition of an artificial tear solution will greatly impact the mass of tear film components that deposit. By simply removing two proteins from the ATS (lactoferrin and immunoglobulin G), lipid deposition significantly decreased. Data has established that the incubation volume (not shown) and lipid component concentrations [75] also affect the amount of lipid deposited. It is known that meibum, tear film, and deposited lipid concentrations and compositions can vary widely between individuals and that diet, medications, systematic diseases, and work environment can influence this deposition [6-9,76,77]. Therefore, it is very difficult to build an in vitro model to fully mimic all of the relationships and interactions that occur in human contact lens wear, so the first step is to begin unraveling the factors that may influence deposition.

When the deposited mass of lipids quantified in this experiment is compared with other in vitro and ex vivo data, it can be seen that differences do exist. In this experiment, after 7 and 20 days of incubation in the ATS solution (+ LF/IgG), balafilcon A lenses deposited 1.80±0.06 and 3.22±0.04 µg of cholesterol and 0.93±0.02 and 1.22±0.07µg of phosphatidylcholine per lens, respectively. Omafilcon A lenses deposited 0.17±0.005 and 0.21±0.02 µg/lens of cholesterol after 7 and 20 days of incubation and similar masses of phosphatidylcholine at the same time points. Much of the other in vitro lipid work completed recently has quantified higher masses of cholesterol and phospholipids (either phosphatidylcholine or phosphatidylethanolamine) depositing on balafilcon A and on conventional hydrogel lens materials such as etafilcon A. In vitro work from Carney et al. [28], Iwata et al. [56], and Pucker et al. [31], all cited higher deposition values than the work presented here. However, these other in vitro studies had one or more of these main differences in their experimental design, which may account for increased deposition of lipids: the use of single lipid incubation solutions, higher concentrations of lipids in the ATS, altered incubation volumes, and replenishment of the ATS with fresh solution during incubation [28,31,56]. All of these factors may explain the higher deposition of cholesterol and phosphatidylcholine.

When the cholesterol deposition results found in this in vitro experiment are compared with recent ex vivo data it is found that results from the balafilcon A material are quite similar. Zhao et al. [57] quantified 4.1–8.2 µg/lens after 30 days of wear (depending on the cleaning solution used) and Saville et al. [78] found 3.9 µg/lens after 30 nights of wear. Saville [78] also examined phosphatidylcholine deposition and quantified 0.019 µg/lens following 30 nights of wear, which is lower than our quantified mass of 1.2 µg/lens on balafilcon A. Many of the recent in vitro and ex vivo studies were not completed with the same silicone hydrogel lens materials, did not include conventional hydrogel lens materials such as omafilcon A, and some of them examined different lipids than those quantified in this experiment.

It is clear that in vitro models do not always directly mimic what happens in vivo. Many times the masses deposited are lower or higher than what is reported in human worn contact lenses. This may be due to the simplicity of the models being used, different ATS compositions and concentrations or an incomplete understanding of all of the interactions and influences that are present. The only way that in vitro models can be improved in their usefulness is to take a more in-depth look at the relationships that are occurring during human contact lens wear and then test and incorporate them into the in vitro models. It may transpire that the success of an in vitro model should not be measured according to the absolute mass deposited during human contact wear, as these values have large variations based on the populations tested, but should be examined to see if the hierarchy of deposition is consistent when comparing different lens materials and if the trends of wear are predictive of human wear. In the end, in vitro models must become more physiologically relevant so that their use can be validated and provide a basis for research and development of new and existing products.

As a first step in developing an in vitro model, the ATS developed in our laboratory has been shown to remain stable throughout incubation periods up to four weeks, the lens parameters show no significant changes following a two week incubation, and deposited lipids are in line with recent ex vivo data. The ATS solution introduced in this paper has the flexibility to be tailored to the individual needs of the specific in vitro experiment and can be used to mimic human worn lens interactions and depositions.

### 

#### Conclusion

This paper has introduced a novel complex artificial tear solution specially designed for in-vial incubations. This solution maintains its own solution parameters and the parameters of the incubating contact lenses constant. This solution characterization is the first step in developing a new in vitro model for contact lens deposition and tear film interactions.

## References

[1] Chamberlain LM, Godek ML, Gonzalez-Juarrero M, Grainger DW (2009). Phenotypic non-equivalence of murine (monocyte-) macrophage cells in biomaterial and inflammatory models.. J Biomed Mater Res A.

[2] De Smet N, Rymarczyk-Machal M, Schacht E (2009). Modification of polydimethylsiloxane surfaces using benzophenone.. J Biomater Sci Polym Ed.

[3] Fischer M, Sperling C, Werner C (2010). Synergistic effect of hydrophobic and anionic surface groups triggers blood coagulation in vitro.. J Mater Sci Mater Med.

[4] Ravenscroft-Chang MS, Stohlman JM, Molnar P, Natarajan A, Canavan HE, Teliska M, Stancescu M, Krauthamer V, Hickman JJ (2010). Altered calcium dynamics in cardiac cells grown on silane-modified surfaces.. Biomaterials.

[5] Subbiahdoss G, Kuijer R, Busscher HJ, van der Mei HC (2010). Mammalian cell growth versus biofilm formation on biomaterial surfaces in an in vitro post-operative contamination model.. Microbiology.

[6] Maïssa C, Guillon M, Girard-Claudon K, Cooper P (2002). Tear lipid composition of hydrogel contact lens wearers.. Adv Exp Med Biol.

[7] Millar TJ, Pearson ML (2002). The effects of dietary and pharmacological manipulation on lipid production in the meibomian and harderian glands of the rabbit.. Adv Exp Med Biol.

[8] Brennan N, Coles M (2000). Deposits and symptomatology with soft contact lens Wear.. ICLC.

[9] Hart DE, Lane BC, Josephson JE, Tisdale RR, Gzik M, Leahy R, Dennis R (1987). Spoilage of hydrogel contact lenses by lipid deposits. Tear-film potassium depression, fat, protein, and alcohol consumption.. Ophthalmology.

[10] Tiffany J (1988). Tear film stability and contact lens wear.. J BCLA.

[11] McCulley JP, Shine W (1997). A compositional based model for the tear film lipid layer.. Trans Am Ophthalmol Soc.

[12] Prydal JI, Artal P, Woon H, Campbell FW (1992). Study of human precorneal tear film thickness and structure using laser interferometry.. Invest Ophthalmol Vis Sci.

[13] Butovich IA, Millar TJ, Ham BM (2008). Understanding and analyzing meibomian lipids–a review.. Curr Eye Res.

[14] Miano F, Calcara M, Millar TJ, Enea V (2005). Insertion of tear proteins into a meibomian lipids film.. Colloids Surf B Biointerfaces.

[15] Miano F, Mazzone M, Giannetto A, Enea V, Mc Cauley P, Bailey A, Winlove PC (2002). Interface properties of simplified tear-like fluids in relation to lipid and aqueous layers composition.. Adv Exp Med Biol.

[16] Millar TJ, Tragoulias ST, Anderton PJ, Ball MS, Miano F, Dennis GR, Mudgil P (2006). The surface activity of purified ocular mucin at the air-liquid interface and interactions with meibomian lipids.. Cornea.

[17] Tragoulias ST, Anderton PJ, Dennis GR, Miano F, Millar TJ (2005). Surface pressure measurements of human tears and individual tear film components indicate that proteins are major contributors to the surface pressure.. Cornea.

[18] Subbaraman LN, Glasier MA, Senchyna M, Sheardown H, Jones L (2006). Kinetics of in vitro lysozyme deposition on silicone hydrogel, PMMA, and FDA groups I, II, and IV contact lens materials.. Curr Eye Res.

[19] Senchyna M, Jones L, Louie D, May C, Forbes I, Glasier MA (2004). Quantitative and conformational characterization of lysozyme deposited on balafilcon and etafilcon contact lens materials.. Curr Eye Res.

[20] Keith DJ, Christensen MT, Barry JR, Stein JM (2003). Determination of the lysozyme deposit curve in soft contact lenses.. Eye Contact Lens.

[21] Berry M, Harris A, Corfield AP (2003). Patterns of mucin adherence to contact lenses.. Invest Ophthalmol Vis Sci.

[22] Keith EO, Boltz M, Gadh R, Ghorsriz R, Mangatt D, Janoff LE (2001). Adhesion of tear proteins to contact lenses and vials.. Biotechnol Appl Biochem.

[23] Bontempo AR, Rapp J (2001). Protein and lipid deposition onto hydrophilic contact lenses in vivo.. CLAO J.

[24] Garrett Q, Garrett RW, Milthorpe BK (1999). Lysozyme sorption in hydrogel contact lenses.. Invest Ophthalmol Vis Sci.

[25] Prager MD, Quintana RP (1997). Radiochemical studies on contact lens soilation. I. Lens uptake of 14C-lysozyme from simple and complex artificial tear solutions.. J Biomed Mater Res.

[26] Boot N, Kok J, Kijlstra A (1989). The role of tears in preventing protein deposition on contact lenses.. Curr Eye Res.

[27] Castillo EJ, Koenig JL, Anderson JM, Jentoft N (1986). Protein adsorption on soft contact lenses. III. Mucin.. Biomaterials.

[28] Carney FP, Nash WL, Sentell KB (2008). The adsorption of major tear film lipids in vitro to various silicone hydrogels over time.. Invest Ophthalmol Vis Sci.

[29] Chow LM, Subbaraman LN, Sheardown H, Jones L (2009). Kinetics of in vitro lactoferrin deposition on silicone hydrogel and FDA group II and group IV hydrogel contact lens materials.. J Biomater Sci Polym Ed.

[30] Suwala M, Glasier MA, Subbaraman LN, Jones L (2007). Quantity and conformation of lysozyme deposited on conventional and silicone hydrogel contact lens materials using an in vitro model.. Eye Contact Lens.

[31] Pucker AD, Thangavelu M, Nichols JJ (2010). In vitro lipid deposition on hydrogel and silicone hydrogel contact lenses.. Invest Ophthalmol Vis Sci.

[32] Mirejovsky D, Patel AS, Rodriguez DD, Hunt TJ (1991). Lipid adsorption onto hydrogel contact lens materials. Advantages of Nile red over oil red O in visualization of lipids.. Optom Vis Sci.

[33] Lorentz H, Heynen M, Jones L (2010). The impact of tear film components on in vitro lipid uptake to silicone hydrogel and hydrogel contact lens materials.. Cont Lens Anterior Eye.

[34] Van Haeringen NJ (1981). Clinical biochemistry of tears.. Surv Ophthalmol.

[35] Lamberts DW. Physiology of the tear film. In: Smolin G, Thoft RA, editors. The cornea. Boston: Little, Brown & Company 1987. p. 38–52.

[36] Giordano R, Costantini S, Vernillo I, Rizzica M (1983). Atomic absorption techniques for the microdetermination of multielements in whole tear film.. Atomic Spectroscopy.

[37] Carney LG, Hill RM (1976). Human tear pH. Diurnal variations.. Arch Ophthalmol.

[38] Chao CC, Vergnes J, Brown S (1983). Fractionation and partial characterization of macromolecular components from human ocular mucus.. Exp Eye Res.

[39] Nicolaides N. Recent finding on the chemical composition of the lipids of steer and human meibomian glands. In: Holly F, editor. The preocular tear film in health, disease, and contact lens wear. Lubbock, Texas: Dry Eye Institute 1986. p. 570–96.

[40] Haberland ME, Reynolds JA (1973). Self-association of cholesterol in aqueous solution.. Proc Natl Acad Sci USA.

[41] Gachon AM, Richard J, Dastugue B (1982-1983). Human tears: normal protein pattern and individual protein determinations in adults.. Curr Eye Res.

[42] Sen DK, Sarin GS (1986). Biological variations of lysozyme concentration in the tear fluids of healthy persons.. Br J Ophthalmol.

[43] van Haeringen NJ, Glasius E (1975). Cholesterol in human tear fluid.. Exp Eye Res.

[44] Prager MD, Quintana RP (1997). Radiochemical studies on contact lens soilation. II. Lens uptake of cholesteryl oleate and dioleoyl phosphatidylcholine.. J Biomed Mater Res.

[45] Stuchell RN, Feldman JJ, Farris RL, Mandel ID (1984). The effect of collection technique on tear composition.. Invest Ophthalmol Vis Sci.

[46] Berta A. Standardization of tear protein determinations: the effects of sampling, flow rate, and vascular permeability. In: Holly F, editor. The preocular tear film in health, disease and contact lens wear. Lubbock, Texas: Dry Eye Institute 1986. p. 418–35.

[47] Fullard RJ (1988). Identification of proteins in small tear volumes with and without size exclusion HPLC fractionation.. Curr Eye Res.

[48] Fullard RJ, Snyder C (1990). Protein levels in nonstimulated and stimulated tears of normal human subjects.. Invest Ophthalmol Vis Sci.

[49] Stathopulos PB, Scholz GA, Hwang YM, Rumfeldt JA, Lepock JR, Meiering EM (2004). Sonication of proteins causes formation of aggregates that resemble amyloid.. Protein Sci.

[50] Castillo EJ, Koenig JL, Anderson JM, Lo J (1985). Protein adsorption on hydrogels. II. Reversible and irreversible interactions between lysozyme and soft contact lens surfaces.. Biomaterials.

[51] Castillo EJ, Koenig JL, Anderson JM (1986). Characterization of protein adsorption on soft contact lenses. IV. Comparison of in vivo spoilage with the in vitro adsorption of tear proteins.. Biomaterials.

[52] Bohnert JL, Horbett TA, Ratner BD, Royce FH (1988). Adsorption of proteins from artificial tear solutions to contact lens materials.. Invest Ophthalmol Vis Sci.

[53] Ho CH, Hlady V (1995). Fluorescence assay for measuring lipid deposits on contact lens surfaces.. Biomaterials.

[54] Bontempo AR, Rapp J (1997). Protein-lipid interaction on the surface of a hydrophilic contact lens in vitro.. Curr Eye Res.

[55] Bontempo AR, Rapp J (1997). Protein-lipid interaction on the surface of a rigid gas-permeable contact lens in vitro.. Curr Eye Res.

[56] Iwata M, Ohno S, Kawai T, Ichijima H, Cavanagh HD (2008). In vitro evaluation of lipids adsorbed on silicone hydrogel contact lenses using a new gas chromatography/mass spectrometry analytical method.. Eye Contact Lens.

[57] Zhao Z, Carnt NA, Aliwarga Y, Wei X, Naduvilath T, Garrett Q, Korth J, Willcox MD (2009). Care regimen and lens material influence on silicone hydrogel contact lens deposition.. Optom Vis Sci.

[58] Tighe B, Panaser A, Franklin V, Campbell D (2009). Tear film lipids: Dynamic composition and clinical performance.. Cont Lens Anterior Eye.

[59] Tomlinson A, Khanal S (2005). Assessment of tear film dynamics: quantification approach.. Ocul Surf.

[60] Nagyová B, Tiffany JM (1999). Components responsible for the surface tension of human tears.. Curr Eye Res.

[61] ISO/CD. Optics and optical instruments - contact lenses - Part 2: Tolerances. ISO #18369–2. Geneva: International Organization for Standardization 2006.

[62] Jones L, Senchyna M, Glasier MA, Schickler J, Forbes I, Louie D, May C (2003). Lysozyme and lipid deposition on silicone hydrogel contact lens materials.. Eye Contact Lens.

[63] Sask KN, Zhitomirsky I, Berry LR, Chan AK, Brash JL (2010). Surface modification with an antithrombin-heparin complex for anticoagulation: studies on a model surface with gold as substrate.. Acta Biomater.

[64] Bodde EW, Boerman OC, Russel FG, Mikos AG, Spauwen PH, Jansen JA (2008). The kinetic and biological activity of different loaded rhBMP-2 calcium phosphate cement implants in rats.. J Biomed Mater Res A.

[65] Kurosawa S, Kamo N, Aizawa H, Muratsugu M (2007). Adsorption of 125I-labeled immunoglobulin G, its F(ab')2 and Fc fragments onto plasma-polymerized films.. Biosens Bioelectron.

[66] Holmberg M, Stibius KB, Larsen NB, Hou X (2008). Competitive protein adsorption to polymer surfaces from human serum.. J Mater Sci Mater Med.

[67] Gilbert TW, Stewart-Akers AM, Badylak SF (2007). A quantitative method for evaluating the degradation of biologic scaffold materials.. Biomaterials.

[68] Jones LC, Tucci M, Frondoza C (2006). Macrophages and fibroblasts respond differently to PMMA particles and mechanical strain.. Biomed Sci Instrum.

[69] Garrett Q, Milthorpe BK (1996). Human serum albumin adsorption on hydrogel contact lenses in vitro.. Invest Ophthalmol Vis Sci.

[70] Bowers RW, Tighe BJ (1987). Studies of the ocular compatibility of hydrogels. White spot deposits–chemical composition and geological arrangement of components.. Biomaterials.

[71] Abbott J, Bowers R, Franklin V, Tighe B (1991). Studies in the ocular compatibility of hydrogels (IV): Observations on the role of calcium in deposit formation.. J BCLA.

[72] Maziarz EP, Stachowski MJ, Liu XM, Mosack L, Davis A, Musante C, Heckathorn D (2006). Lipid deposition on silicone hydrogel lenses, part I: quantification of oleic Acid, oleic Acid methyl ester, and cholesterol.. Eye Contact Lens.

[73] Greiner JV, Glonek T, Korb DR, Booth R, Leahy CD (1996). Phospholipids in meibomian gland secretion.. Ophthalmic Res.

[74] Greiner JV, Glonek T, Korb DR, Leahy CD (1996). Meibomian gland phospholipids.. Curr Eye Res.

[75] Walther H, Lorentz H, Kay L, Heynen M, Jones L. The Effect of in vitro Lipid Concentration on Lipid Deposition on Silicone Hydrogel and Conventional Hydrogel Contact Lens Materials. British Contact Lens Association Conference 2011; Manchester, UK.: Poster# 20.

[76] Dougherty JM, Osgood JK, McCulley JP (1991). The role of wax and sterol ester fatty acids in chronic blepharitis.. Invest Ophthalmol Vis Sci.

[77] Tiffany JM (1978). Individual variations in human meibomian lipid composition.. Exp Eye Res.

[78] Saville JT, Zhao Z, Willcox MD, Blanksby SJ, Mitchell TW (2010). Detection and quantification of tear phospholipids and cholesterol in contact lens deposits: the effect of contact lens material and lens care solution.. Invest Ophthalmol Vis Sci.

